# 基于二代测序的微小残留病检测发现儿童急性B淋巴细胞白血病克隆演变1例报告并文献复习

**DOI:** 10.3760/cma.j.cn121090-20240527-00190

**Published:** 2024-12

**Authors:** 娇 常, 玉娇 贾, 浩旭 王, 本泉 戚, 小矜 蔡, 琦 孙, 晓凡 竺, 志坚 肖, 慧君 王

**Affiliations:** 1 中国医学科学院血液病医院（中国医学科学院血液学研究所），血液与健康全国重点实验室，国家血液系统疾病临床医学研究中心，细胞生态海河实验室，天津 300020 State Key Laboratory of Experimental Hematology, National Clinical Research Center for Blood Diseases, Haihe Laboratory of Cell Ecosystem, Institute of Hematology & Blood Diseases Hospital, Chinese Academy of Medical Sciences & Peking Union Medical College, Tianjin 300020, China; 2 天津医学健康研究院，天津 301600 Tianjin Institutes of Health Science, Tianjin 301600, China

## Abstract

微小残留病（MRD）是血液肿瘤患者经治疗取得完全缓解后体内残存微量肿瘤细胞的状态，也是评价治疗效果和预测疾病复发的重要标志物。本文回顾性分析了1例多次复发的急性B淋巴细胞白血病患儿的临床诊治及MRD监测过程，并进行相关文献复习。在本例患儿中，基于二代测序（NGS）的Ig重排MRD检测与传统MRD检测方法相比可更准确地评估MRD水平，更早地提示疾病复发，从而指导临床及时采取干预措施。此外，NGS-MRD还能发现疾病克隆演变，为进一步探究疾病发展的内在因素提供了新思路。

## Abstract (en)

Minimal residual disease（MRD）, a crucial biomarker for assessing efficacy and predicting recurrence, refers to residual tumor cells remaining in the body of patients with hematological malignancies who achieved complete remission after treatment. This study aimed to conduct a retrospective analysis of the clinical diagnosis, treatment, and MRD monitoring of a pediatric patient with multiple acute B-lymphocytic leukemia relapses, alongside a review of relevant literature. In this case, Ig rearrangement based on next-generation sequencing（NGS）was more accurate in assessing the MRD level, compared with the traditional method of MRD detection, indicating the risk of earlier relapse and guided interventions in time. Additionally, NGS-MRD detected clonal evolution, providing new ideas to further investigate the intrinsic factors of disease development.

急性淋巴细胞白血病（ALL）是儿童时期最常见的恶性肿瘤，目前儿童ALL患者5年总生存（OS）率已超过90％[1]，但仍有部分患儿表现为难治或复发，预后不良。患者体内极低水平的微小残留病（MRD）可能是疾病复发的根源，因此MRD监测在患者的治疗过程中尤为重要。研究发现，传统检测手段显示MRD阴性的患者仍会复发。基于二代测序（NGS）的淋系肿瘤Ig/TCR基因克隆性重排MRD检测因其高灵敏度逐渐成为新一代MRD检测方法，近年来在ALL的MRD监测中有较大优势[2]–[4]。本文报道1例基于NGS的Ig重排MRD检测发现克隆演变并预测疾病复发的儿童B-ALL。

## 病例资料

患儿，男，11岁，2021年12月因“憋气逐渐加重”就诊于中国医学科学院血液病医院，外周血涂片示原始淋巴细胞94％；染色体检查示体质性异常inv（2）（q21q23）；血液系统融合基因筛查阴性。流式细胞术免疫表型检测可见两群异常B淋巴细胞，一群前B（Pre B）表型占47.3％，另一群偏成熟表型占43.2％。NGS-Ig重排检测到2种IGH克隆性重排（IGH-A、IGH-B），MRD水平为9.85×10^−1^。综合上述检查结果患儿诊断为B-ALL。2021年12月按照CCCG-ALL-2020方案予地塞米松进行窗口期治疗，予VDLP方案（长春新碱+柔红霉素+培门冬酶+泼尼松）诱导化疗，化疗过程中患儿出现持续反复高热、多部位感染及脏器多部位病变导致化疗中断。2022年3月流式细胞术MRD检测可见89.07％异常B淋巴母细胞。2022年5月应用贝林妥欧单抗靶向治疗，后续复查骨髓细胞形态学、流式细胞术均未见异常。2022年7月骨髓涂片可见原始幼稚淋巴细胞12％，流式细胞术示异常B淋巴母细胞3.24％，考虑疾病复发。2022年7、8、9月继续贝林妥欧单抗治疗，期间骨髓细胞形态学、流式细胞术均未见异常。2022年11月复查骨髓涂片示完全缓解骨髓象，流式细胞术未见异常B淋巴细胞，此时NGS-Ig重排仍检测到初诊时的两种IGH克隆性重排残留，MRD水平为1.12×10^−2^。2022年11月骨髓细胞形态学示幼稚淋巴细胞5％；流式细胞术示异常B淋巴母细胞4.61％，提示复发趋势，此时NGS-Ig重排不仅检测到初诊时的2种IGH克隆性重排有残留，而且检出1种新的IGH克隆性重排（IGH-New），MRD水平为8.17×10^−2^。2022年12月进行嵌合抗原受体T细胞（CAR-T细胞）单采，后予低剂量化疗（长春地辛+地塞米松+维奈克拉）。2023年1月骨髓细胞形态学示全面复发，流式细胞术回报异常B淋巴母细胞39.75％，NGS-Ig重排回报MRD水平为8.69×10^−1^，除检测到IGH-A、IGH-B、IGH-New克隆性重排有残留外，还检测出1种新的IGKDE克隆性重排（IGKDE-New）。2023年1月进行CAR-T细胞治疗。2023年1月和2月复查骨髓MRD，流式细胞术均为阴性，NGS-Ig重排示MRD水平分别为1.09×10^−4^和1.08×10^−3^。2023年2月起予FC方案（氟达拉滨+环磷酰胺）清除淋巴细胞后再次回输CAR-T细胞。2023年3月患儿于院外复查血常规：WBC 15.07×10^9^/L，HGB 121 g/L，PLT 45×10^9^/L，考虑复发。2023年3月本院骨髓细胞形态学示复发骨髓象，流式细胞术示异常B淋巴母细胞83.90％，NGS-Ig重排检测示MRD水平为9.75×10^−1^，除检测到IGH-A、IGH-B、IGH-New、IGKDE-New克隆性重排有残留外，还检测出1种新的IGL克隆性重排（IGL-New），予地塞米松、长春新碱和抗感染治疗，但患者WBC增长极快，病情危重，家属充分了解病情后要求出院。

## 讨论及文献复习

在许多回顾性和前瞻性研究中，MRD是与ALL复发和总生存（OS）期高度相关的预后指标，MRD阴性的患者复发率更低，OS期更长[5]–[7]。ALL患者传统的MRD评估方法主要包括多参数流式细胞术（MFC）、实时定量聚合酶链反应（RQ-PCR）检测特异性融合基因或特异性Ig/TCR基因克隆性重排。但研究表明，传统方法检测MRD阴性的患者仍有25％甚至更高的复发率[8]–[10]，可能是由于患者体内存在低于常规检测水平的MRD，因此需要利用更加灵敏的检测手段进行MRD监测。

基于NGS的Ig/TCR重排检测可对待测样本BCR/TCR的CDR3区域进行多重PCR扩增，再使用高通量测序技术对扩增产物进行检测，通过分析测序结果可在治疗前样本中鉴定肿瘤相关克隆性重排，进而在后续治疗样本中检测肿瘤相关克隆是否存在及其数量，从而评估患者的MRD水平。基于NGS检测Ig/TCR重排的灵敏度可达1×10^−6^，与MFC和RQ-PCR相比，检测灵敏度提高了1～2个对数级[11]–[12]。

近年来的研究表明，无论是成人还是儿童ALL，基于NGS的Ig/TCR重排MRD检测较MFC或RQ-PCR均表现出显著优势。Short等[2]回顾性分析了74例接受一线治疗的成人ALL患者（65例B-ALL，9例T-ALL），MFC显示MRD阴性的70个缓解样本中，32个样本NGS检测显示MRD阳性，且处于完全缓解，与MFC-MRD阴性患者相比，NGS-MRD阴性患者的5年累积复发率更低（0％对29％），5年OS率更高（90％对68％）。同样，Wu等[3],[13]分别在92例儿童B-ALL和43例儿童T-ALL患者中发现部分MFC检测MRD阴性的样本NGS检测为阳性，而MFC检测MRD阳性的样本NGS检测均为阳性。此外，Kotrova等[14]和Svaton等[4]分别在76例和432例儿童ALL患者中发现NGS-MRD可纠正qPCR-MRD的假阴性和假阳性结果，从而更准确地进行疾病风险分层和预测疾病复发。造血干细胞移植是治疗高风险ALL患者的有效手段之一，移植前后MRD评估对预测患者预后具有重要意义。Pulsipher等[15]报道，COG ASCT0431研究中56例儿童B-ALL患者移植前及移植后（尤其是移植后早期）进行的NGS-MRD检测均较MFC-MRD检测更准确地预测疾病复发和生存。除此之外，Pulsipher等[16]在2021年进一步探究了低于NGS-MRD检测下限的MRD水平与临床预后的相关性，显示19例NGS-MRD水平<1×10^−6^的ALL患者中，14例复发，4例接受了额外治疗，只有1例持续缓解，提示NGS-MRD水平低于检测限仍具有重要意义。

在本例患儿中，利用NGS-Ig重排检测在治疗前样本中对IGH、IGH-DJ、IGK、IGKDE、IGL进行多重PCR扩增和高通量测序，鉴定出2种IGH克隆性重排，即IGH-A和IGH-B。在后续MRD监测过程中，NGS-Ig重排较MFC灵敏度更高，能更准确地评估MRD水平，尤其是在两次疾病复发前（2022年11月和2023年2月），在传统方法检测MRD为阴性时，NGS-Ig重排均检出较高水平的MRD残留（图1），可更早地提示疾病复发可能，便于及时对疾病进行治疗。此外，NGS-Ig重排还能发现克隆演变（图2），本例患儿在疾病第2次复发初期、第2次全面复发及第3次复发时利用NGS-Ig重排均检测到新克隆，为进一步探究疾病发展的内在原因提供了新思路。在本病例中，患儿共经历了3次疾病复发，但由于样本量不足，第1次疾病复发（2022年7月）前后未能利用NGS-Ig重排进行MRD监测。各阶段NGS-Ig重排检测结果见表1，各克隆性重排信息见表2。

**图1:**
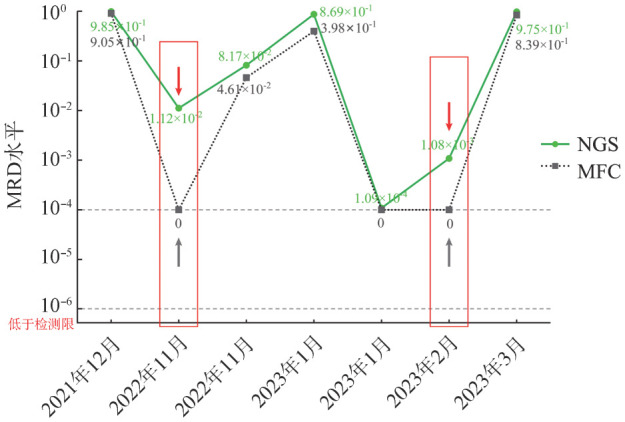
二代测序（NGS）与多参数流式细胞术（MFC）检测本例急性B淋巴细胞白血病患儿微小残留病（MRD）水平

**图2:**
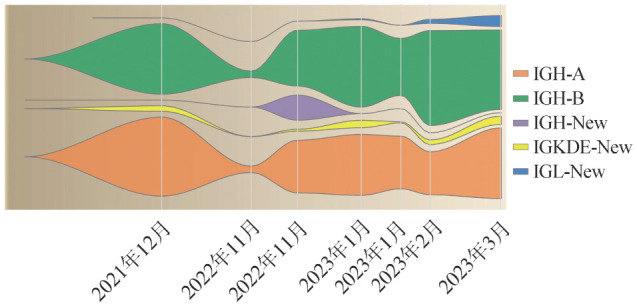
二代测序检测本例急性B淋巴细胞白血病患儿Ig克隆性重排占比变化

**表1:** 本例急性B淋巴细胞白血病患儿微小残留病（MRD）监测结果

MRD监测时间	骨髓细胞形态学	流式细胞术	基于二代测序的-Ig重排（克隆占比^a^/有核细胞占比^b^）
2021年12月	原始淋巴细胞94％	47.30％异常B淋巴母细胞（Pre B-ALL表型）；43.20％异常B淋巴母细胞（偏成熟表型）	IGH-A（52.00％/52.00％）、IGH-B（46.45％/46.45％）
2022年11月	治疗后骨髓象	未见异常B淋巴母细胞	IGH-A（4.19％/0.55％）、IGH-B（4.33％/0.57％）
2022年11月	原始淋巴细胞5％	4.61％异常B淋巴母细胞	IGH-A（34.65％/3.22％）、IGH-B（36.75％/3.41％）、IGH-New（16.53％/1.54％）^c^
2023年1月	原始淋巴细胞70％	39.75％异常B淋巴母细胞	IGH-A（40.20％/37.41％）、IGH-B（53.24％/49.54％）、IGH-New（0.01％/0.01％）、IGKDE-New（4.94％/0.49％）^c^
2023年1月	治疗后骨髓象	未见异常B淋巴母细胞	IGH-A（34.68％/0.01％）、IGH-B（37.56％/0.01％）、IGH-New（0/0）、IGKDE-New（0.81％/0）
2023年2月	治疗后骨髓象	未见异常B淋巴母细胞	IGH-A（28.27％/0.03％）、IGH-B（62.49％/0.07％）、IGH-New（0/0）、IGKDE-New（3.04％/0）
2023年3月	原始淋巴细胞84％	83.90％异常B淋巴母细胞	IGH-A（46.52％/46.52％）、IGH-B（52.33％/52.33％）、IGH-New（0.01％/0.01％）、IGKDE-New（5.46％/0.32％）、IGL-New（7.27％/0.34％）^c^

**注** ^a^该克隆性重排在同类型所有重排中所占比例；^b^含有该克隆性重排的细胞在总有核细胞中所占比例；^c^新的克隆性重排

**表2:** 本例急性B淋巴细胞白血病患儿二代测序Ig克隆性重排信息

克隆性重排	CDR3序列	V/D/J基因
IGH-A	TGTGCAAGAGATGGGGGGAGAGATAGTAGTACCCCTAAACGACTTGACTACTACTACGGTATGGACGTCTGG	IGHV6-1/IGHD2/IGHJ6
IGH-B	TGTGCAAAAGGCCTCCGGGCTGCTACTCAAACTACTACTACTACGGTATGGACGTCTGG	IGHV3-9/IGHD2-2/IGHJ6
IGH-New	TGTGCAAAAGGACTCCGGGCTGCTACTCAAACTACTACTACTACGGTATGGACGTCTGG	IGHV3-9/IGHD2-2/IGHJ6
IGKDE-New	TATTACGTCTGCAGAGTAAGAATTTTCCTCCTGAGCCCTAGTGGCAGCCCAGGGCGACTCCTCATGAGT	IGKV7-3/–/Kde
IGL-New	TGCAGCTCATATGCAGGCAGCAACCAGGGAATGGGTCTTC	IGLV2-8/–/IGLJ1

**注** –：无

总之，基于NGS的Ig重排MRD检测作为新的分子生物学技术，不仅具有通量高、灵敏度高等优势，可更准确地评估患者MRD水平、评价治疗效果并预测疾病复发，而且能为临床提供更多信息，包括肿瘤克隆性重排多样化信息、IGH体细胞高突变率、克隆演变及免疫组库信息等，逐渐成为MRD评估的手段之一，但该技术仍需进一步标准化。
